# A case of gastric antral vascular ectasia in which PuraStat, a novel self‐assembling peptide hemostatic hydrogel, was effective

**DOI:** 10.1002/deo2.183

**Published:** 2022-11-10

**Authors:** Yoshitsugu Misumi, Miharu Takeuchi, Maiko Kishino, Yoshimichi Kudo, Kouichi Nonaka

**Affiliations:** ^1^ Department of Digestive Endoscopy Tokyo Women's Medical University Hospital Tokyo Japan; ^2^ Department of Pediatric Cardiology and Adult Congenital Cardiology Tokyo Women's Medical University Hospital Tokyo Japan

**Keywords:** endoscopic hemostasis, gastric antral vascular ectasia, gastric hemorrhage, PuraStat, self‐assembling peptide hydrogel

## Abstract

Gastric antral vascular ectasia (GAVE) is a gastric hemorrhagic disease associated with chronic liver disease. Argon plasma coagulation is widely used to control gastrointestinal bleeding due to GAVE. Although argon plasma coagulation is a relatively safe endoscopic procedure, it is not suitable in some cases, such as in patients with pacemakers. We report a case of GAVE in which PuraStat, a novel self‐assembling peptide hemostatic hydrogel, was effective. The patient was a 55‐year‐old man who had undergone Fontan surgery for tricuspid regurgitation more than 20 years prior. He developed hepatic cirrhosis as a complication following Fontan surgery. During upper gastrointestinal endoscopy to examine the cause of the progression of anemia and black stool, bleeding from GAVE was observed; PuraStat was applied to stop the bleeding. Postoperatively, the black stool disappeared, and his hemoglobin levels improved. Upper gastrointestinal endoscopy was performed 13 days after the surgery; the density of the capillaries in the antrum was significantly decreased, and a clear trend toward disappearance was observed. Therefore, the application of PuraStat may be useful in the treatment of GAVE.

## INTRODUCTION

Gastric antral vascular ectasia (GAVE), also known as watermelon stomach, is a disease in which telangiectasia radiates radially in the antrum and often occurs in the background of chronic liver disease, chronic renal failure, and autoimmune diseases.^1^ GAVE has been reported to account for approximately 4% of cases of non‐variceal upper gastrointestinal bleeding. Endoscopic hemostasis using argon plasma coagulation (APC) is widely performed when GAVE results in anemia.^2^ Argon plasma coagulation, a non‐contact electrocoagulation technique, is a relatively safe endoscopic hemostasis technique that requires only a shallow ablation depth. However, complications such as perforation and intestinal stricture during the healing process have been reported.^3^ GAVE associated with liver cirrhosis often requires multiple treatment sessions; however, frequent treatments can place a physical and economic burden on patients. PuraStat (3D Matrix, Tokyo, Japan) is a novel self‐assembling peptide hemostatic hydrogel that can be used endoscopically.^4^ The peptide molecule in PuraStat consists of a repeated sequence of arginine, alanine, aspartic acid, and alanine amino acids and has a β‐sheet structure.^5^ When this peptide molecule encounters body fluids, such as blood, it is neutralized. Additionally, when electrolytes such as sodium or potassium are supplied, it forms fibers and becomes a peptide hydrogel. This peptide hydrogel coats the bleeding point and physically stops the bleeding.^6^ Excluding spurting bleeding, general cases of gastrointestinal bleeding are indicated for hemostasis using PuraStat. Furthermore, PuraStat has been reported to have a wound‐healing effect in intestinal injury in a rat model.^7^ Clinically, PuraStat has been reported to be useful in promoting the healing of radiation‐induced proctitis.^8^ We report a case of GAVE in which PuraStat was effective in treating gastric bleeding.

## CASE REPORT

**FIGURE 1:**
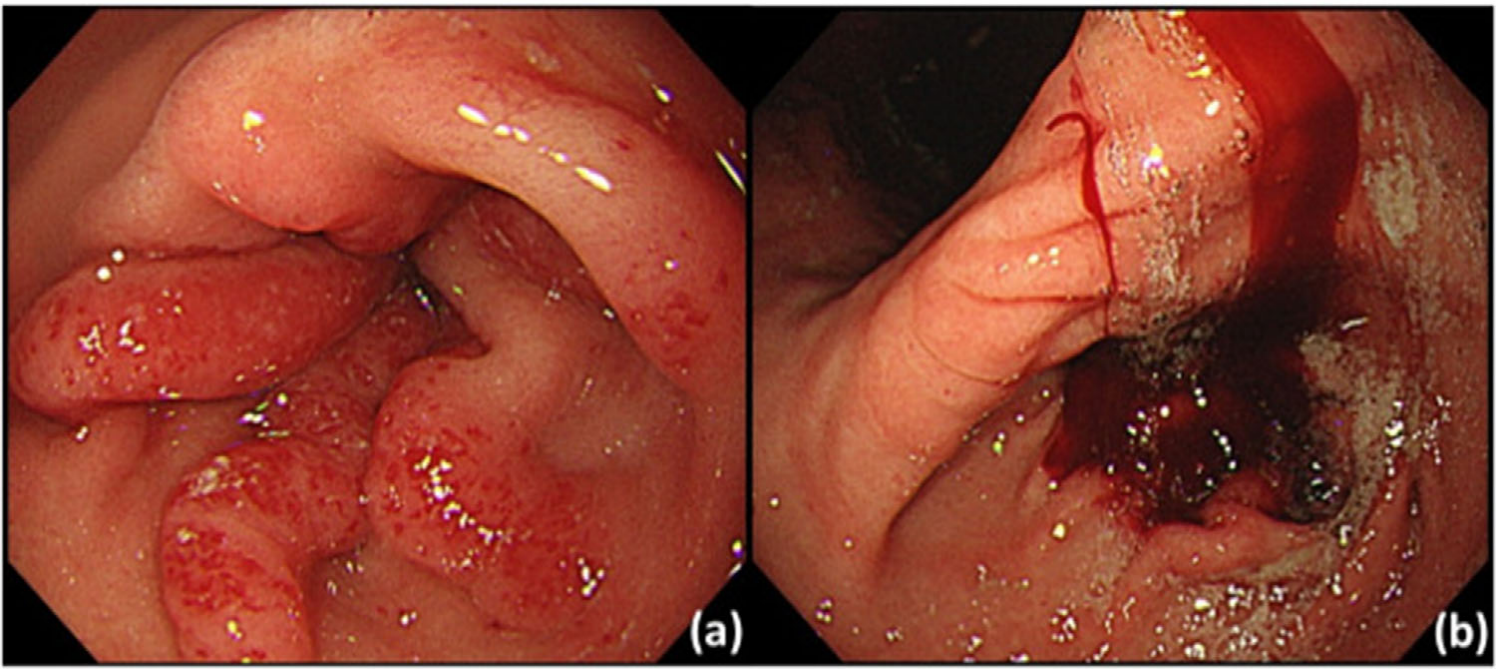
(a) Radial linear telangiectasia in the antrum, typical of watermelon stomach. (b) Clot adhesion and oozing were observed immediately after insertion.

The patient was a 55‐year‐old man who had undergone Fontan surgery for tricuspid atresia more than 20 years prior. He was diagnosed with liver cirrhosis due to Fontan‐associated liver disease and was admitted to the hospital for control of retained ascites and leg edema. He also had a history of chronic atrial fibrillation, was taking warfarin, and had an implanted pacemaker for sick sinus syndrome. A blood sample at admission showed pancytopenia including thrombocytopenia associated with liver cirrhosis (white blood cell count: 2990 per μl, red blood cell count: 2.53×10^6^ per μl, hemoglobin (Hb): 7.7 g/dl, platelets: 7.2×10^4^ per μl). Upper gastrointestinal hemorrhage was suspected due to findings of black stool before admission and a drop in Hb concentration from the patient's usual 10–7.7 g/dl at admission. However, on admission, the black stool subsided, and it was concluded that there was no active hemorrhage; hence, the patient's condition was maintained with daily blood transfusion and oral administration of a proton pump inhibitor. Oral administration of warfarin was also continued. With blood transfusion, Hb concentration temporarily rose to 9.8 g/dl; however, a small amount of black stool appeared again, and Hb dropped to 8.3 g/dl, indicating active upper gastrointestinal hemorrhage. Thus, esophagogastroduodenoscopy (EGD) was performed after informed consent was obtained. EGD revealed findings of a watermelon stomach in the antrum, and the patient was diagnosed with GAVE (Figure 1a). A blood clot adhered to the antrum immediately after insertion, and spontaneous bleeding occurred from the GAVE (Figure 1b).

**FIGURE 2:**
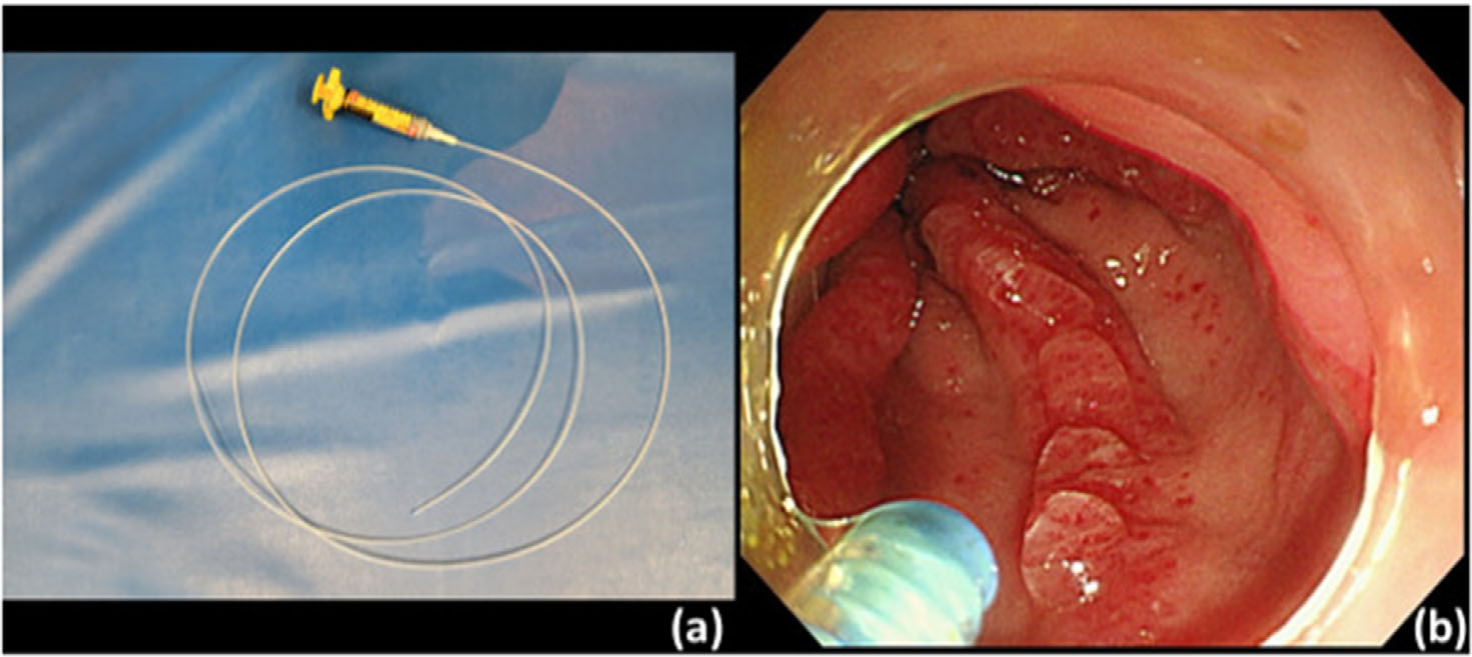
(a) Dedicated catheter for PuraStat. (b) A catheter was used to create a small gel bulge on top of the telangiectasia.

Endoscopic hemostasis using APC was considered; however, there were concerns regarding interference with the pacemaker. Moreover, our facility was not equipped with bipolar hemostatic forceps that could avoid interference with the pacemaker. Therefore, endoscopic hemostasis using PuraStat, a novel self‐assembling peptide hemostatic hydrogel, was attempted. A dedicated catheter with a dull tip was used for the PuraStat application (Figure 2a). A small gel bulge was applied to the dilated telangiectasia causing the oozing hemorrhage, and primary hemostasis was obtained. In addition, a total of 5 ml PuraStat solution was applied in the same way to the reddened telangiectasias that did not exhibit bleeding (Figure 2b).

**FIGURE 3:**
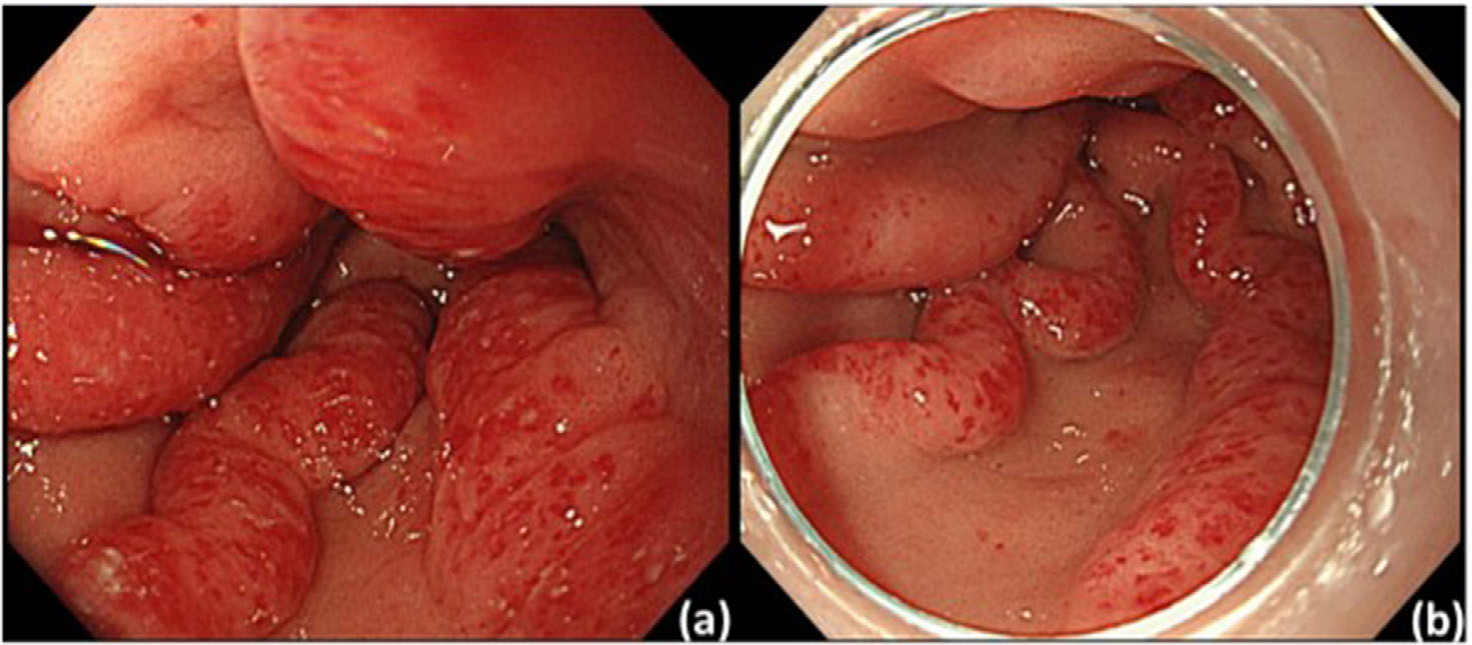
Comparison of esophagogastroduodenoscopy findings on day 1 (a) and day 13 (b). A clear decrease in the density of telangiectasia and the disappearance of redness was observed.

EGD was performed on day 13 to evaluate the patient's condition before he was transferred to another hospital. In EGD findings, telangiectasia showed a decreasing trend overall, and no spontaneous bleeding was observed (Figure 3). On the other hand, his Hb value continued to decrease immediately after applying PuraStat. However, a blood test on day 11 showed an increase in Hb (Table 1). Following that, from day 2 onward, no black stool was observed. Due to the patient's poor cardiac condition, Hb concentration below 8.0 g/dl was set as the standard for conducting blood transfusion; however, no decrease in Hb requiring blood transfusion was observed before his hospital transfer on day 23. His Hb concentration in the final blood test taken at the end of the observation period at our hospital was 8.7 g/dl. After his hospital transfer, as of postoperative day 36, no black stool was observed and additional blood transfusion was not required. His Hb concentration on postoperative day 36 was 9.2 g/dl.

**TABLE 1:** Changes in hemoglobin over time. Although there was a time lag from the application of PuraStat, the progression of anemia was slowed

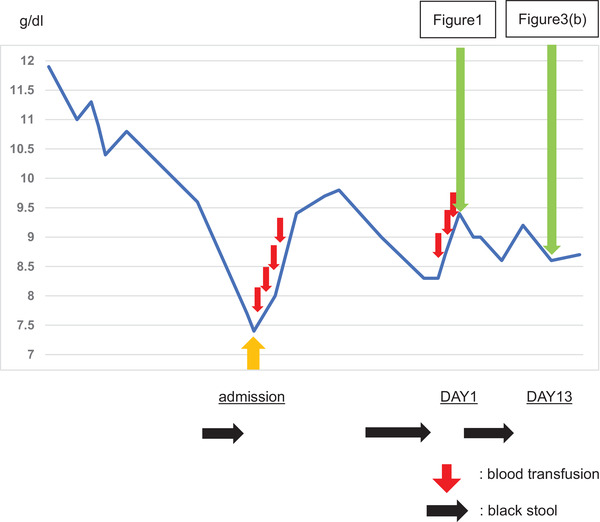

## DISCUSSION

We experienced a case of GAVE that was successfully treated with the endoscopic application of PuraStat, a novel self‐assembling peptide hemostatic hydrogel. APC is most widely performed for gastrointestinal bleeding due to GAVE, and the use of other endoscopic treatments, such as endoscopic band ligation, is also known. Further, it has been reported that APC in combination with hemostatic powder is effective in treating intractable GAVE.^9^ APC is a relatively safe endoscopic hemostatic surgery and rarely causes complications; however, it is not completely safe due to reports of postoperative perforation and stenosis.^3^ APC can also cause electromagnetic interference to pacemakers.^10^ In this case, it was difficult to perform APC. On the other hand, the endoscopic application of PuraStat is a safe and simple procedure with an extremely low risk of complications; it involves simply pressing a dedicated catheter with a blunt tip onto the mucous membrane.

Conventional surgical hemostatic agents use human‐derived fibrin or bovine‐derived collagen as raw materials. However, the peptides that comprise the raw materials of PuraStat are manufactured by chemical synthesis, eliminating animal‐derived substances; thus, it can be said that there is no risk of infection with the hepatitis C virus, and so forth.^5^ PuraStat is originally a local hemostatic agent, and primary hemostasis of bleeding due to GAVE was obtained in this case as well. In addition to the hemostatic effect, PuraStat has also been reported to have a wound‐healing effect.^7^, ^8^ In this case, 13 days after the PuraStat application, the density of telangiectasia decreased significantly overall, and the redness of each blood vessel was visibly improved. Here, it was presumed that the hemostatic and wound‐healing effects acted locally, and the endoscopic improvement of telangiectasia was observed. The endoscopic application of PuraStat can be a safe and simple new treatment option for GAVE.

## CONFLICT OF INTEREST

None.

## ETHICS STATEMENT

All procedures followed were performed in accordance with the ethical standards of the 1964 Declaration of Helsinki and its later amendments.
